# A new compartmental method for the analysis of liver FDG kinetics in small animal models

**DOI:** 10.1186/s13550-015-0107-1

**Published:** 2015-06-11

**Authors:** Sara Garbarino, Valentina Vivaldi, Fabrice Delbary, Giacomo Caviglia, Michele Piana, Cecilia Marini, Selene Capitanio, Iolanda Calamia, Ambra Buschiazzo, Gianmario Sambuceti

**Affiliations:** Dipartimento di Matematica, Università di Genova, via Dodecaneso 35, 16146 Genova, Italy; CNR - SPIN Genova, via Dodecaneso 33, 16146 Genova, Italy; CNR Institute of Molecular Bioimaging and Physiology Milan, section of Genova, c/o Nuclear Medicine, Largo Rossana Benzi 10, 16132 Genova, Italy; Nuclear Medicine Unit, IRCCS AOU San Martino-IST, Università di Genova, Largo Benzi 10, 16132 Genova, Italy; Dipartimento di Scienze della Salute, Università di Genova, Largo Benzi 10, 16132 Genova, Italy

**Keywords:** Compartmental analysis, Liver physiology, Dual input, FDG-PET, Metformin

## Abstract

**Background:**

Compartmental analysis is a standard method to quantify metabolic processes using fluorodeoxyglucose-positron emission tomography (FDG-PET). For liver studies, this analysis is complex due to the hepatocyte capability to dephosphorylate and release glucose and FDG into the blood. Moreover, a tracer is supplied to the liver by both the hepatic artery and the portal vein, which is not visible in PET images. This study developed an innovative computational approach accounting for the reversible nature of FDG in the liver and directly computing the portal vein tracer concentration by means of gut radioactivity measurements.

**Methods:**

Twenty-one mice were subdivided into three groups: the control group ‘CTR’ (*n* = 7) received no treatment, the short-term starvation group ‘STS’ (*n* = 7) was submitted to food deprivation with free access to water within 48 h before imaging, and the metformin group ‘MTF’ (*n* = 7) was treated with metformin (750 mg/Kg per day) for 1 month. All mice underwent a dynamic micro-PET study for 50 min after an ^18^F-FDG injection. The compartmental analysis considered two FDG pools (phosphorylated and free) in both the gut and liver. A tracer was carried into the liver by the hepatic artery and the portal vein, and tracer delivery from the gut was considered as the sole input for portal vein tracer concentration. Accordingly, both the liver and gut were characterized by two compartments and two exchange coefficients. Each one of the two two-compartment models was mathematically described by a system of differential equations, and data optimization was performed by applying a Newton algorithm to the inverse problems associated to these differential systems.

**Results:**

All rate constants were stable in each group. The tracer coefficient from the free to the metabolized compartment in the liver was increased by STS, while it was unaltered by MTF. By contrast, the tracer coefficient from the metabolized to the free compartment was reduced by MTF and increased by STS.

**Conclusions:**

Data demonstrated that our method was able to analyze FDG kinetics under pharmacological or pathophysiological stimulation, quantifying the fraction of the tracer trapped in the liver or dephosphorylated and released into the bloodstream.

## Background

Hepatic glucose metabolism is of relevance in a number of clinical syndromes and, in particular, in diabetes [1-5]. Several authors previously evaluated the potential of positron emission tomography (PET) with ^18^F-fluorodeoxyglucose (FDG) in this setting, both in humans and animals [2,3]. However, a standard method has not been developed so far because of the peculiarities of liver function and anatomy. From the physiological viewpoint, hepatocytes can dephosphorylate glucose-6-phosphate (G6P) to deliver glucose back into the bloodstream. This feature also applies to FDG-6-phosphate (FDG-6P) preventing the accumulation kinetics typical of most tissues and nicely suited to compartmental analysis. From the anatomical viewpoint, the dual blood supply from the hepatic artery (HA) and portal vein (PV) further hampers the definition of tracer delivery to hepatic tissue [6]. Actually, limiting the analysis to the arterial input function (IF) inevitably causes systematic underestimation of tracer distribution in the liver [7-11]. Accordingly, several attempts have been made to estimate the dual-input IFs from dynamic PET data [8,12,13]. However, no method successfully solved this problem so far, since the PV is hard to recognize in PET images and suffers from a relevant partial-volume effect related to vessel size and respiratory movements.

According to these limitations, several authors tried to indirectly predict the PV tracer concentration by the arterial IF. More precisely, one approach applied ad hoc algorithms to compute the PV IF as a heuristic modification of the arterial one [5,9]. A second approach directly and invasively sampled the PV IF [3-5] and then determined the dual IF as a weighed superposition of the two activities [10,14-18]. However, both methods are model-dependent and difficult to apply in a noninvasive setting, while their potential under non-physiological conditions has been scarcely explored.

**Figure 1:**
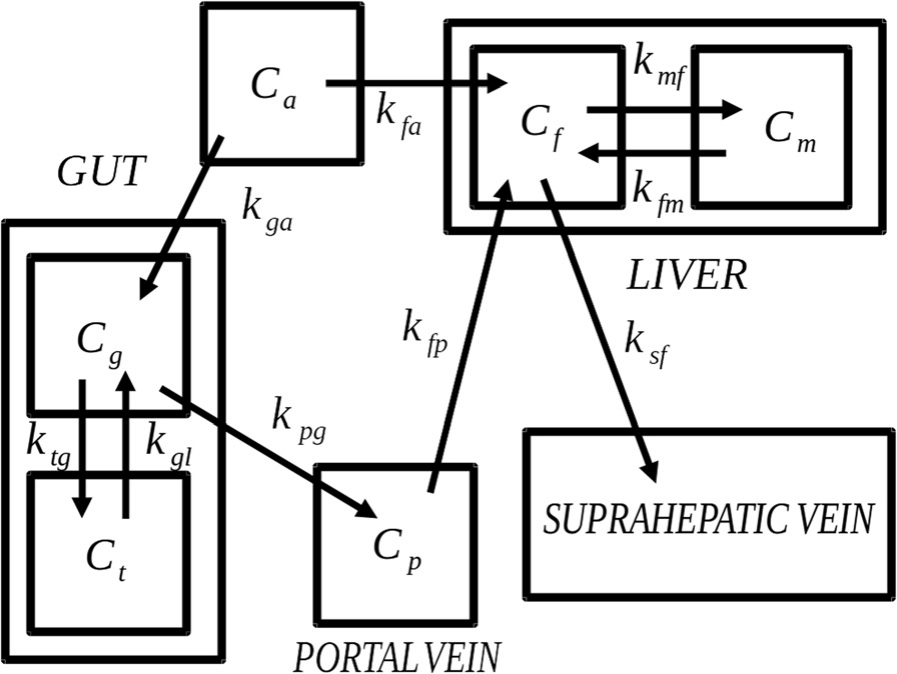
Compartmental model for the gut and liver. The ‘portal vein’ compartment utilizes *k*_*pg*_ to compute *C*_*p*_.

To overcome these limitations, we coupled the capability of micro-PET to provide FDG time activity curves (TACs) in virtually all organs with the formulation of a novel compartmental model (see Figure 1) for the hepatic system using the following three descriptors of tracer kinetics in the liver:

1. The time-concentration curve in the arterial system.
2. The time-concentration curve in the liver.
3. The time-concentration curve in the whole gut as the source of the PV blood.

We used this information as input data of an iterative algorithm [19] that reduces a compartmental model in which gut tracer concentration is used to compute the PV IF, while liver activity is used to describe tracer kinetics in the hepatic system. In the present study, we aimed at validating this algorithm by testing its performance in mice treated with interventions able to blunt or enhance liver G6P dephosphorylation and glucose delivery.

## Methods

### Reagents

Metformin was provided by Sigma-Aldrich (St. Louis, MO, USA). FDG was produced in-house, according to standard methodology. Daily quality controls always documented adequate standards and, in particular, a radiochemical purity ≥98%.

### Animal models

All animal experiments were reviewed and approved by the Licensing and Ethical Committee of the IRCCS San Martino IST, Genova, Italy, and by the Italian Ministero della Salute. A total of 21 six-week-old BALB/c female mice (Charles River Laboratories, Italy) were housed under specific pathogen-free conditions and subdivided into three different groups according to the treatment preceding the imaging study. The first group of controls (CTR) included seven untreated animals that did not receive any treatment and were kept under standard conditions for the whole study duration. The short-term starvation group (STS) included seven mice that were submitted to food deprivation with free access to water within 48 h before imaging. Finally, the last group (MTF) included seven mice treated with metformin for the month preceding the imaging test. In these animals, the drug was administered diluted in autoclaved drinking water at a concentration of 3 mg/mL as to account for a dose of 750 mg/Kg per day [11].

### Animal preparation and image processing

**Figure 2:**
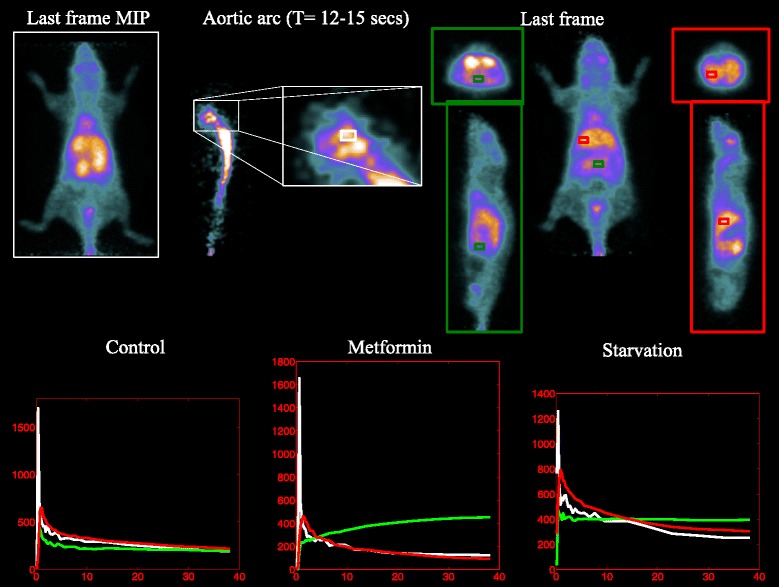
Typical ROIs and TACs of the aortic arc (white), gut (green), and liver (red).

In vivo imaging was performed according to a protocol validated in our lab [11]. To ensure a steady state of substrate and hormones governing glucose metabolism, all animals were studied after 6 h of fasting. Mice were weighed, and anesthesia was induced by intraperitoneal administration of ketamine/xylazine (100 and 10 mg/Kg, respectively). Serum glucose level was measured, and animals were positioned on the bed of a dedicated micro-PET system (Albira, Bruker, USA) whose two-ring configuration permits to cover the whole animal body in a single bed position. A dose of 3 to 4 MBq of FDG was then injected through a tail vein, soon after the start of a list mode acquisition lasting 50 min. Acquisition was reconstructed using the following framing rate: 10 × 15 s, 5 × 30 s, 2 × 150 s, 6 × 300 s, and 1 × 600 s. PET data were reconstructed using a maximum likelihood expectation maximization (MLEM) method. Thereafter, each image dataset was reviewed by an experienced observer who recognized three regions of interest (ROIs) encompassing the aortic arc, gut, and liver, respectively (see Figure 2). The same figure also shows the TACs computed from the three ROIs in the case of animals belonging to the analyzed groups (CTR, STS, MTF). We are aware that the determination of the arterial IF is a challenging task in the case of mice. To accomplish it, for each animal model, we have first viewed the tracer first pass in cine mode. Then, in a frame where the left ventricle was particularly visible, we have drawn a ROI in the aortic arc and maintained it for all time points. Therefore, in this study, we used the resulting TAC as arterial IF. We recognize that this procedure may be affected by partial volume effects in the PET data; however, we expect this kind of error to be systematic for all datasets and therefore to affect the results in a way which is essentially independent of the experiment. Cine mode representation was used also to obtain the gut and liver TACs, since especially kidneys and bladder often display a biphasic curve due to tracer filtration-accumulation and possible voiding preventing their recognition in the last frame. For example, in the gut case, a ROI was drawn analyzing in cine mode all 27 frames of acquisition; the ROI volumes ranged between 15 and 30 μL and were placed systematically in the anterior abdomen, paying care to exclude contamination from the liver, spleen, kidney, and large vessels throughout all frames.

### Compartmental modeling of FDG kinetics

Our approach for the definition of the dual IF was based on processing a complete set of ROIs drawn on the aortic arc, gut, and liver. In particular, we aimed to describe the gut by means of a compartmental model with one arterial IF and one output directly delivering the tracer in the PV blood. This latter concentration was then used as the venous IF in a second compartmental model focused on the liver. Since both the gut and liver express G-6P-phosphatase, they were described by means of two functional compartments that account for the significant exchange between trapped and free tracer, i.e., between FDG-6P and FDG. This compartmental system is described in Figure 1.

### Gut subsystem

This subsystem was considered to have one arterial IF, one output function to the PV, and two compartments: the free tracer in the gut (denoted with *g*) and the trapped FDG-6P (denoted with *t*). The mathematical model at the basis of our compartmental analysis relied on the balance of the tracer activities (i.e., concentrations per unit volume) between the different compartments. This balance leads to a number of ordinary differential equations, which are a standard in this framework. In the specific case of the gut tracer kinetics, the compartmental model is made of the following two differential equations with vanishing initial data:

1 $$ {\dot{C}}_g=-\left({k}_{tg}+k{}_{pg}\right){C}_g+{k}_{gt}{C}_t+{k}_{ga}{C}_a $$

2 $$ {\dot{C}}_t={k}_{tg}{C}_g-{k}_{gt}{C}_t. $$

In these equations, *C*_*g*_, *C*_*t*_, and *C*_*a*_ represent the tracer concentrations in the free compartment *g*, in the phosphorylated compartment *t*, and in the arterial blood *a*, respectively; the superposed dot indicates differentiation with respect to time, and the coefficients *k*_*ij*_ (measured in min^−1^) denote the rate coefficients to the target compartment *i* from the source compartment *j*. Equations (1) and (2) can be formally solved to obtain the analytical expressions of *C*_*g*_ and *C*_*t*_. We observe that such expressions depend on the tracer coefficients that at this stage of the solution process are still unknown.

In order to describe the output of the gut subsystem, we introduced a further compartment *p*, anatomically represented by the PV. Assuming that in such a compartment the blood flow is constant (which implies *k*_*pg*_ = *k*_*fp*_), activity conservation leads to a third differential equation for the concentration *C*_*p*_:

3 $$ {\dot{C}}_p={k}_{pg}{C}_g-{k}_{pg}{C}_p. $$

Also, in the case of Equation (3), the analytical solution can be formally determined and depends on the kinetics parameter *k*_*pg*_.

### Liver subsystem

According to standard results [17], also the liver can be described as a two-compartment model, one consisting of non-metabolized, free, tracer (compartment *f*), and one consisting of phosphorylated, metabolized tracer (compartment *m*), where, as in the case of the gut, dephosphorylation is explicitly allowed. Tracer inputs through the HA and PV (compartments *a* and *p*, respectively) are modeled in an independent manner, i.e., there is no mixing of blood from the two vessels before entrance into the liver. On the contrary, possible heterogeneities in perfusion source may occur as to consider the most general input model. Accordingly, exchange coefficients for the HA blood (*k*_*fa*_) and the PV blood (*k*_*fp*_) are considered as independent. On the other hand, FDG delivery to the PV blood is considered to occur only from the gut, tracer exchange throughout the vein is negligible, and thus, the whole tracer amount entered into the PV is available for liver uptake.

We now denote with *C*_*f*_ and *C*_*m*_ the concentration of the free and metabolized FDG pools, respectively, with *k*_*sf*_ the rate coefficient from the free compartment to the venous efflux to the suprahepatic vein *s*, *k*_*mf*_ the exchange coefficient from the FDG to the FDG-6P pool, and *k*_*fm*_ the exchange coefficient for the inverse process. Then, the usual assumption on the conservation of activities provides:

4 $$ {\dot{C}}_f=-\left({k}_{mf}+{k}_{sf}\right){C}_f+{k}_{fm}C{}_m+{k}_{fa}{C}_a+{k}_{pg}{C}_p $$

and

5 $$ {\dot{C}}_m={k}_{mf}{C}_f-{k}_{fm}{C}_m. $$

Equations (4) and (5) can be again formally solved to obtain the analytical expressions of *C*_*f*_ and *C*_*m*_. We observe again that such expressions depend on the tracer coefficients that at this stage of the solution process are still unknown.

### Data optimization

Micro-PET data provide information on the overall concentrations in ROIs drawn on the gut and liver throughout the whole acquisition. Therefore, denoting with ${\tilde{C}}_{\mathrm{gut}}$ and ${\tilde{C}}_{\mathrm{liver}}$ such experimental concentrations, we can write the following two equations for the micro-PET data:

6 $$ {\tilde{C}}_{\mathrm{gut}}={C}_g+{C}_t $$

7 $$ {\tilde{C}}_{\mathrm{liver}}-V\left(0.11{C}_a+0.89{C}_p\right)=\left(1-V\right)\left({C}_f+{C}_m\right) $$

where the numerical coefficients 0.11 and 0.89 indicate the rate of arterial and venous contributions to the hepatic blood content *V* per unit volume [8,17]. Further, we assumed for *V* the physiologically sound value of 0.3 [9]. In principle, these values may change between the different groups; therefore, we made the same computation for different pairs of values (0.15 to 0.85, 0.25 to 0.75, 0.5 to 0.5, respectively). The mean values of the tracer coefficients did not change significantly while the corresponding uncertainties increased with respect to the choice 0.11 to 0.89.

In order to numerically solve Equations (6) and (7) and therefore to determine the tracer coefficients, we applied, separately and in cascade, a regularized multi-dimensional Newton algorithm [19], where a nice trade-off between the numerical stability of the problem solution and an appropriate fitting of the measured data were obtained by means of an optimized selection of the regularization parameter. To this aim, we first observed using simulations that the regularized Newton algorithm is rather robust with respect to the choice of the regularization parameter. In fact, in the case of a synthetic dataset, there exists a unique value of the regularization parameter that minimizes the distance between the reconstructed and ground-truth tracer coefficient vector. For all simulations performed, this value had an order of magnitude of around 10^4^, and tuning such value in the range of 10^3^ to 10^5^ changed the reconstructed coefficients of less than 0.5%. In the case of experimental data, for each mouse, we applied a discrepancy approach: we chose as optimal value of the regularization parameter the value for which the discrepancy between the experimental data and the data predicted by the regularized solution coincided with the uncertainty on the measurement [20]. This uncertainty was computed by assuming that the noise on the activity is Poisson and the values of the regularization parameter we obtained were around 10^4^, as in the simulated cases. We are aware that the Poisson assumption on the uncertainty does not account for correlation and other effects induced by the reconstruction algorithm; however, the robustness of regularization guarantees for the reliability of this approach.

From a computational viewpoint, the codes implementing the reduction of these compartmental models are extremely fast (less than 1 min for each analysis). Further, in order to assess the numerical robustness of the approach, for each animal model we have perturbed 50 times the PV TAC (obtained from the reduction of the gut subsystem) with 50 different Poisson random components, and accordingly, we have computed the tracer coefficients for the liver. For each coefficient, the standard deviation corresponding to the mean value over the several runs of the algorithm represents a reliable measure of the robustness of the approach with respect to uncertainties in the computation of the PV TAC. We found out that all standard deviations were below 4% of the mean values.

## Results

### Experimental protocol

The experiments were completed in all animals, and no side effects occurred at the drug dosage used. Body weight was stable, and glycemia was reduced only in the STS mice (Table 1). In agreement with our previous experience [21], the response of whole body glucose metabolism was characterized by a relative reduction in FDG clearance and whole body glucose consumption in both MTF and STS animals. Similarly [11], this systemic effect was paralleled by a peculiar response of tracer distribution in the different investigated organs. In fact, MTF treatment was followed by an increase in gut tracer retention and thus in higher gut average standardized uptake value (SUV) with respect to control (2.49 ± 0.97 vs 1.07 ± 0.36, *p* < 0.01 vs CTR). On the contrary, liver FDG accumulation was eventually similar, independently of experimental conditions (Table 1).

**Table 1:** Means and standard deviations for weight, glycemia, and SUV over the three sets

	**Weight (** ***g*** **)**	**Glycemia (mg/dl)**	**SUV gut**	**SUV liver**
CTR	24.4 ± 1.8	131.1 ± 12.1	1.07 ± 0.36	1.07 ± 0.33
MTF	23.9 ± 1.3	89.1 ± 18.6	2.49 ± 0.97	1.03 ± 0.31
STS	23.7 ± 1.6	54.0 ± 24.3	1.51 ± 0.39	1.17 ± 0.27

SUV data have been measured at the last experimental frame.

### Estimation of the PV TAC

**Figure 3:**
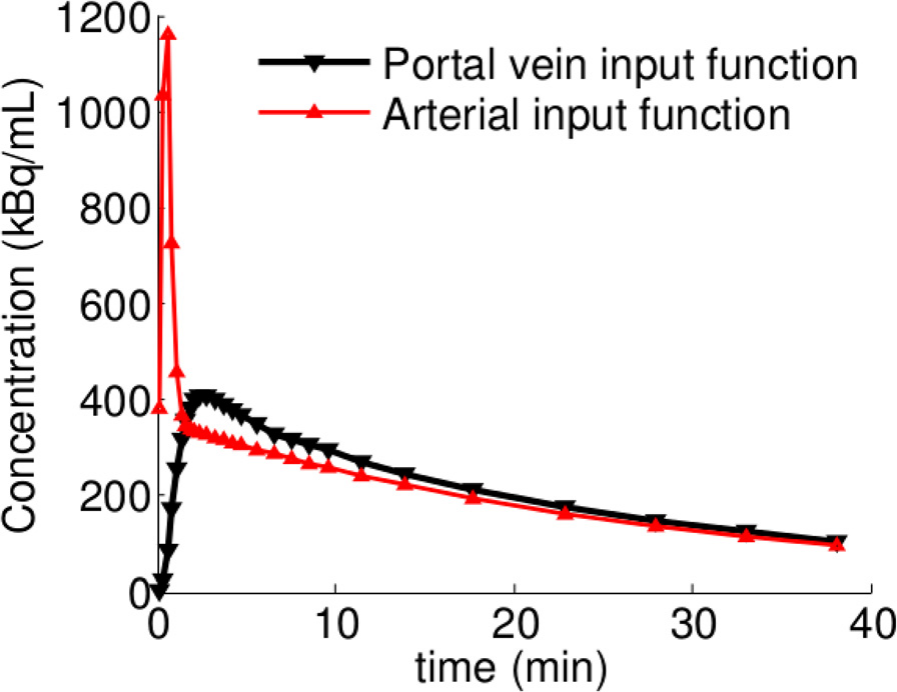
Comparison between the HA and PV tracer concentrations in one mice.

While in each animal the arterial TAC was obtained experimentally, the TAC in the PV was the result of the computational analysis. More precisely, we solved Equation (3) and sampled the time range according to typical acquisition times for ‘Albira’ (10 × 15 s, 5 × 30 s, 2 × 150 s, 6 × 300 s, 1 × 600 s). This curve (see Figure 3) well fitted with expectations based on previous literature [6,8].

### Effects on liver FDG kinetics

**Figure 4:**
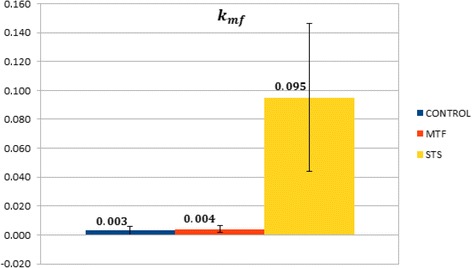
Comparison between the values of *k*_*mf*_ in the metabolized and the free compartment. Each bar represents the mean over a specific set of mice, while the black line represents standard deviation.

In our compartmental analysis, rate constants of FDG handling in the liver were indicated as *k*_*mf*_ and *k*_*fm*_; they represent, respectively, the exchange coefficients from the free compartment to the phosphorylated one and its reciprocal. According to the values given in Table 2 and Figure 4, *k*_*mf*_ in the liver was left virtually unaltered by MTF (0.004 ± 0.003 min^−1^, *p* = ns vs CTR), while it was markedly increased by STS (0.095 ± 0.051 min^−1^, *p* < 0.05 vs the CTR and MTF group), as expected as a consequence of the fasting condition.

**Table 2:** Means and standard deviations for the gut and liver kinetics parameters over the three sets

	***k***_***fa***_	***k***_***mf***_	***k***_***sf***_	***k***_***fm***_	***k***_***fp***_	***k***_***ga***_	***k***_***tg***_	***k***_***gt***_
CTR	2.180 ± 0.568	0.003 ± 0.003	2.51 ± 0.51	0.154 ± 0.059	2.291 ± 0.530	1.109 ± 0.331	0.036 ± 0.029	0.047 ± 0.029
MTF	2.112 ± 0.420	0.004 ± 0.003	2.478 ± 0.389	0.041 ± 0.026**	2.068 ± 0.757	0.902 ± 0.305	0.118 ± 0.073	0.029 ± 0.012
STS	2.384 ± 0.689	0.095 ± 0.051*	3.801 ± 1.650	0.563 ± 0.221**	2.135 ± 0.646	0.963 ± 0.373	0.060 ± 0.008	0.181 ± 0.10

With *, we indicate all comparisons with *p* < 0.05; with **, all comparisons with *p* < 0.01. All the *k*_*ij*_ are measured in min^−1^.

**Figure 5:**
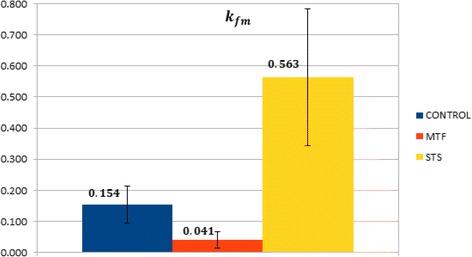
Comparison between the values of *k*_*fm*_ in the metabolized and the free compartment. Each bar represents the mean over a specific set of mice, while the black line represents standard deviation.

The rate coefficient *k*_*fm*_ describes dephosphorylation of FDG-6-P inside the liver. According to Table 2 and Figure 5, this process was markedly reduced by MTF (0.041 ± 0.026 min^−1^ vs 0.154 ± 0.059 min^−1^, *p* < 0.01 vs CTR). On the contrary, *k*_*fm*_ was increased by STS to 0.5632 ± 0.221 min^−1^ (*p* < 0.01 vs CTR). This last result gives evidence to the occurrence of an increase of this coefficient, expressing the rate exchange between the metabolized and the free compartment, as a natural consequence of a significant caloric restriction.

We further observe that a Patlak analysis of the same data did not allow us to differentiate the three groups, since we found statistically comparable Patlak slopes (1.09 ± 0.11 for CTR, 1.08 ± 0.12 for MTF, and 1.11 ± 0.24 for STS). Table 2 also reports an increase of *k*_*sf*_ (3.801 ± 1.650 min^−1^ vs 2.51 ± 0.51 min^−1^, *p* = 0.07 vs CTR) and an increase of *k*_*fa*_ in the STS models (2.384 ± 0.689 min^−1^ vs 2.180 ± 0.568 min^−1^, *p* = 0.07 vs CTR), which corresponds to a significant release of FDG from the mice liver to the bloodstream and a less significant input increase from the blood to the liver, induced by starvation. The effect of MTF treatment on these coefficients was negligible.

Finally, no statistically significant change in the values of *k*_*fp*_ for the three groups was observed.

## Discussion

This study introduces a computation-based, model-dependent method for the use of FDG to study glucose kinetics in the liver. The main advantage and novelty of this approach are that it is able to determine the dual input typical of liver physiology just by means of computation, thus avoiding: 1) the use of invasive catheter-based measurement procedures and 2) the major drawback of methods relying on the sole arterial IF, which are not able to account for the intrinsic variability of perfusion and glucose consumption in splanchnic organs. Other attempts to compute the PV IF have been performed ([8] and references within), but those cases rely on a heuristic modification of the arterial IF which is obtained once for all and applied to all animal models. On the contrary, our method is intrinsically model-dependent, since it utilizes compartmental analysis of the gut to directly estimate the PV IF, which, in its turn, is combined with the arterial TAC (obtained via ROIs on the aortic arc) to perform the compartmental analysis of the liver. From a numerical viewpoint, this task was accomplished applying the regularized Newton algorithm to the inverse problems associated to the multi-dimensional Cauchy problems describing the two-compartment gut and liver systems, respectively. In this setting, the computation of this algorithm, which is rather similar to other optimization algorithms applied in compartmental analysis [22-25], is rather effective, since the matrix differentiation step required at some stage can be realized analytically, thus, avoiding time consuming numerical differentiation. The advantages of this technique are the ability to correctly identify the response of rate constants to experimental interventions able to modify hepatic glucose metabolism and a notable robustness with respect to data optimization. The robustness of the method has been tested by computing the tracer coefficients for the liver in the case of different perturbed values of the PV TAC for each animal model. The corresponding standard deviations were always below 4% of the mean values, showing the notable numerical robustness of the numerical process.

### Pathophysiological considerations

The liver plays a crucial role in the maintenance of glycemic levels despite a large variability in glucose consumption in the different body tissues. To this purpose, liver metabolism is regulated as to counterbalance the hyperglycemic response to food intake and to adapt serum glucose concentration to whole body needs under fasting periods. This buffer function is of pivotal importance since - once entered in the cytosol of almost all mammalian cells - glucose is sequestered by its phosphorylation to G6P. The irreversible nature of this reaction also applies to FDG whose accumulation as FDG-6P accounts for the potential of this tracer in the study of brain and tumor metabolism [26].

Coherently with its role in maintaining metabolic homeostasis, this simplified model does not apply to hepatocytes whose G6P content - either newly synthetized or coming from glycogen breakdown - can be hydrolyzed to P_i_ and glucose by G6Pase. This reaction replenishes cytosol content of glucose to be released into the bloodstream. In agreement with this homeostatic role, liver G6Pase catalytic function is downregulated by insulin response to feeding [12] while it is markedly enhanced by the high glucagon levels occurring during fasting periods [13].

Our results favorably compared with this well-validated biochemical model. In the present study, effect of feeding was not tested, as this condition abolishes the steady-state of serum glucose and insulin needed for an accurate compartmental analysis of liver metabolism. Nevertheless, STS increased rate constant *k*_*fm*_ (as an index of FDG-6P dephosphorylation to FDG) about threefold with respect to 6 h of fasting, indicating a reliability of our measurements.

This finding was confirmed by the data obtained under the opposite condition. To this purpose, we studied animals exposed to prolonged treatment with high doses of MTF, the most widely used drug in the treatment of type 2 diabetes. MTF exerts its anti-hyperglycemic effect mainly decreasing hepatic gluconeogenesis via AMP-dependent protein kinase (AMPK) activation through the upstream kinase liver kinase B1 (LKB1) [7]. More importantly, this molecular response also downregulates or even silences the expression of all gluconeogenic enzymes and mostly of G6Pase [27]. Again, our approach to liver FDG kinetics nicely agreed with this mechanism of action. In fact, treatment with high MTF doses profoundly reduced *k*_*fm*_ values, indicating a reduced rate of G6P hydrolysis as expected on the basis of a reduced G6Pase abundance. In this line, our data extend previous studies documenting an increase in hepatic glucose uptake under MTF treatment in subjects with type 2 diabetes [3-5]. This finding has been usually attributed to a direct increase in glucokinase function induced by the improved metabolic control. Our data indicate that, besides the enhancement in retention mechanism, MTF also decreases glucose and FDG output by inhibiting G6Pase activity. Even more interestingly, the response of *k*_*fm*_ to both STS and MTF was paralleled by a relative invariance of *k*_*fp*_ and *k*_*sf*_ in the three mice groups analyzed, demonstrating a similar blood input (PV) and output (SV) in the liver vein system.

Interestingly, in STS models, the value of the rate constant from arterial blood to the liver free compartment (*k*_*fa*_) was increased, probably due to the low glucose concentration in the blood during caloric restriction and the consequent increased demand by the liver.

## Conclusions

In conclusion, our data demonstrated the effectiveness of an innovative computational method to analyze the liver FDG kinetics under pharmacological or pathophysiological stimuli. Although we used a rather complex compartmental model characterized by nine unknowns, the regularized Newton algorithm was able to estimate all unknowns with a satisfactory degree of robustness. This permitted to quantify the fraction of tracer trapped in the liver or dephosphorylated and released into the bloodstream and thus conveyed to the different tissues.

### Consent

Informed consent was obtained from all individual participants included in the study.
